# Supplementary material for: Pre-Treatment and Preoperative Neutrophil-to-Lymphocyte Ratio Predicts Prognostic Value of Glioblastoma: A Meta-Analysis
Source: Brain Sci. 2022 May 21;12(5):675. doi: 10.3390/brainsci12050675 (PMC9139478; doi:10.3390/brainsci12050675)
Supplement: Supplementary file 1 [file brainsci-12-00675-s001.zip › brainsci-1701923-supplementary.pdf]

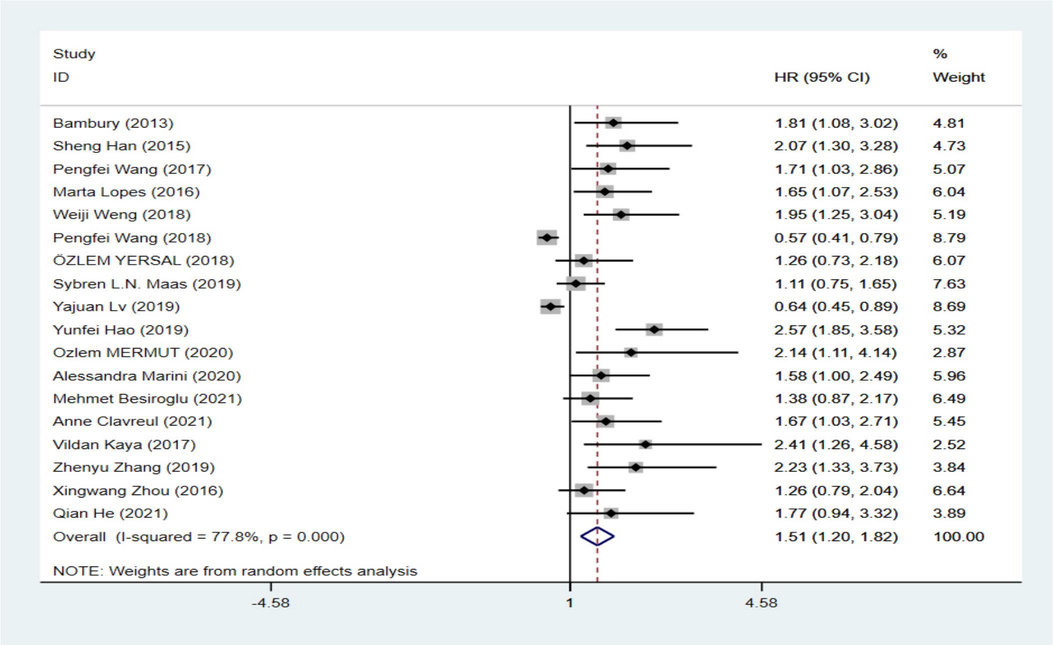

Figure S1. Cancellation of 1 secondary surgical study，Forest plot illustrating the relationship between NLR and OS in glioblastoma patients.

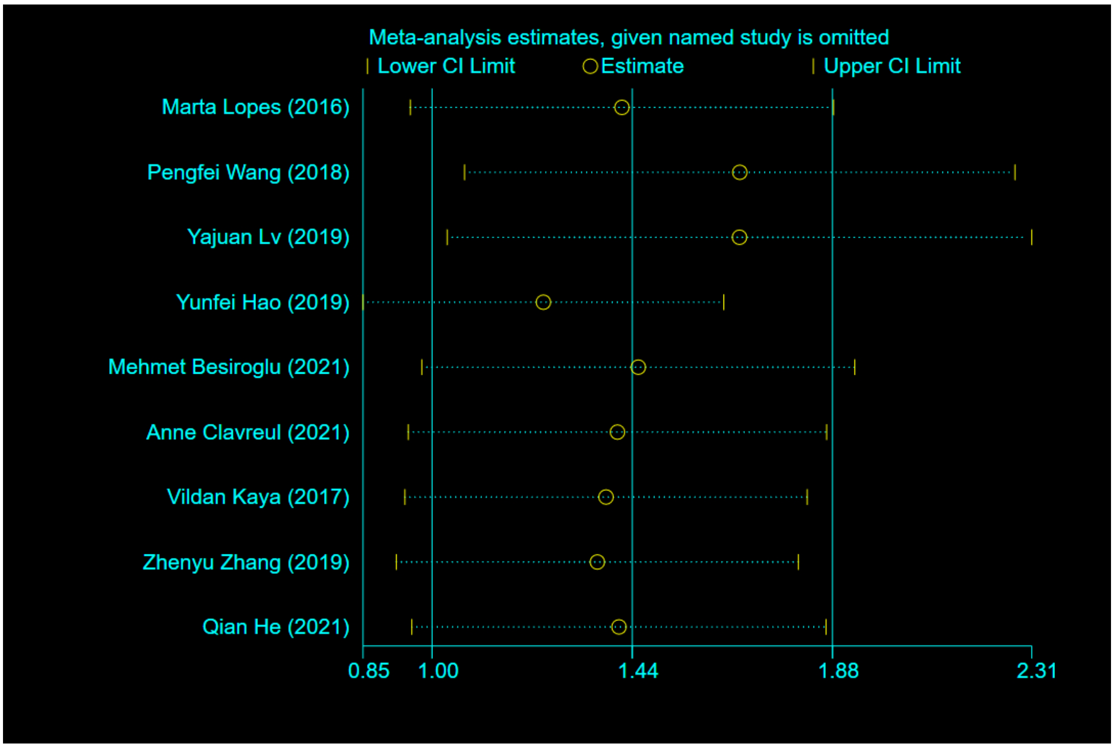

Figure S2. Subgroup sensitivity analysis for truncation values other than4.
